# Dataset on child nutritional status and its socioeconomic determinants in Nonno District, Ethiopia

**DOI:** 10.1016/j.dib.2017.07.007

**Published:** 2017-07-11

**Authors:** Messay Mulugeta, Haregewoin Mirotaw, Bechaye Tesfaye

**Affiliations:** a College of Development Studies, Addis Ababa University, Ethiopia; b Ethiopian Investment Commission, Ethiopia

**Keywords:** Child nutritional status, Socioeconomic, Stunting, Wasting, Underweight, Nonno

## Abstract

This data article presents child nutritional status and its socioeconomic determinants in Ethiopia with special reference to Nonno District, Oromia Region. As recommended by WHO (2006) [1], the nutritional status in this data article is based on three indices (height-for-age, weight-for-height and weight-for-age) for the children in this survey. The data was obtained from four hundred eight (408) households selected by using simple random sampling procedure. The data article shows that the overall prevalence of child malnutrition in the community was high with 46.3% of the children stunted, 41.9% underweight and 11.5% wasted. Moreover, the dataset presents family size, parental education, wealth status of the household, preceding birth interval, antenatal care (ANC) attendance, disease and sanitation are vital determinants of child malnutrition in the area.

**Specifications Table**

**Table:** 

Subject area	Nutrition
More specific subject area	Determinants and nutritional status of children
Type of data	Table and text file
How data was acquired	Household questionnaire survey and child anthropometric measurement
Data format	Analyzed
Experimental factors	We made use of standard hanging balance and height board
Experimental features	Anthropometric data was exported to ENA for SMART 2011 software and converted into Z-scores of the indices: height-for-age, weight-for-height and weight-for-age. Socioeconomic data was analyzed by using to SPSS Version 20 software.
Data source location	Nonno district (8^°^15′N-8^°^40′N and 37^°^20′ E-37^°^35′E), Ethiopia
Data accessibility	The data is with this article

**Value of the data**

• The data presented in this article gives a general picture on the socioeconomic and child nutritional status of the rural community in Ethiopia.
• The data provides information on the socioeconomic determinants of child malnutrition in Ethiopia with special reference to Nonno district.
• The data are important for interventions related to improve child nutritional status in Ethiopia.
• The data are important for planning for socioeconomic development of the rural households in Ethiopia.
• The data can be used by researchers and academicians for further researches and for references.

## 1 Data

This data article presents the nutritional status of children and the socioeconomic determinants of child nutritional status in Nonno district, Ethiopia. Table 1 presents demographic and socioeconomic characteristics while Table 2 portrays housing and hygienic conditions of the surveyed households. Table 3, Table 4 present data on stunting, underweight and wasting. Table 5, Table 6, Table 7, Table 8 present data on the association between the determinant variables and stunting, underweight and wasting.

**Table 1:** Demographic and socioeconomic characteristics of the households.

**Characteristics**	**Number of respondents**	**Percent**
Head of household		
Male	390	95.6
Female	18	4.4
Current marital status of head of household		
Married	391	95.8
Divorced	10	2.5
Separate	6	1.5
Widowed	1	0.2
Family/household size		
≤5	179	43.9
6+	229	56.1
Number of under-five children in the household		
1	160	39.2
2	181	44.4
≥3	67	16.4
Paternal education		
No education	188	46.1
Primary and above	219	53.7
Maternal education		
No education	349	85.5
Primary and above	59	14.5
Paternal occupation		
Farmer	384	94.1
Other	23	5.6
Maternal occupation		
House wife	374	91.7
Other	34	8.3
Mothers age		
≤25	195	47.8
26–34	165	40.4
≥35	48	11.8
Mothers age at first birth		
≤20	378	92.6
≥21	30	7.4
Livestock animals		
Yes	350	85.8
No	58	14.2
Own farm land		
<2 ha	109	26.7
≥2 ha	262	64.2
No	37	9.1
Radio		
Yes	102	25
No	306	75
Ethnicity		
Oromo	388	95.1
Other	20	4.9
Religion		
Muslim	379	92.9
Other	29	7.1

**Table 2:** Environmental characteristics of the households.

**Characteristics**	**Number of respondents**	**Percent**
**Types of houses**		
Tukul/thatched	160	39.2
Corrugated iron sheet	248	60.8
**Type of floor of house**		
Soil	401	98.3
Cement or brick	7	1.7
**Number of rooms**		
1	128	31.4
2	173	42.4
≥3	107	26.2
**Latrine**		
No latrine	112	27.5
Private/wooden slab	279	68.4
Shared/wooden slab	17	4.2
**Presence of window**		
Yes	109	26.7
No	299	73.3
**Main source of drinking water**		
Public pump	357	87.5
Pond	41	10
Unprotected spring	10	2.5
**Waste disposal**		
Open field	233	57.1
In a pit	97	23.8
Composting	61	15
Burning	17	4.2
**Separate room for kitchen**		
Yes	155	38
No	253	62
**Separate room for livestock**		
Yes	147	36
No	261	64
**Type of fuel**		
Wood and animal dung	408	100

**Table 3:** Prevalence of stunting based on height-for-age z-scores and by sex.

**Stunting**	**All*n*=408**	**Boys*n*=214**	**Girls*n*=194**
Prevalence of stunting (<−2 z-score)	(189) 46.3%	(105) 49.1%	(84) 43.3%
Prevalence of moderate stunting (<−2 z-score and ≥−3 z-score)	(136) 33.3%	(74) 34.6%	(62) 32.0%
Prevalence of severe stunting (<−3 z-score)	(53) 13.0%	(31) 14.5%	(22) 11.3%

**Table 4:** Prevalence of underweight based on weight-for-age z-scores by sex.

**Underweight**	**All*n*=408**	**Boys*n*=214**	**Girls*n*=194**
Prevalence of underweight (<−2 z-score)	(171) 41.9%	(98) 45.8%	(73) 37.6%
Prevalence of moderate underweight (<−2 z-score and ≥−3 z-score)	(117) 28.7%	(67) 31.3%	(50) 25.8%
Prevalence of severe underweight (<−3 z-score)	(54) 13.2%	(31) 14.5%	(23) 11.9%

**Table 5:** Prevalence of acute malnutrition based on weight-for-height z-scores and by sex.

Wasting	All*n*=408	Boys*n*=214	Girls*n*=194
Prevalence of global malnutrition (<−2 z-score)	(47) 11.5%	(27) 12.6%	(20) 10.3%
Prevalence of moderate malnutrition (<−2 z-score and ≥−3 z-score)	(35) 8.6%	(20) 9.3%	(15) 7.7%
Prevalence of severe malnutrition (<−3 z-score)	(12) 2.9%	(7) 3.3%	(5) 2.6%

**Table 6:** Association between stunting and selected socioeconomic variables.

Selected variables	Stunting	
	Stunted frequency (%)	Not stunted frequency (%)
Household family size		
≤5	41 (10.05)	138 (33.82)
≥6	148 (36.27)	81 (19.85)
No. of under-five children		
1	58 (14.22)	102 (25.00)
2	84 (20.59)	97 (23.77)
≥3	47 (11.52)	20 (4.90)
Paternal education		
No education	123 (30.22)	65 (15.97)
Primary and above	65 (15.97)	154 (37.84)
Wealth of household		
Low	122 (29.9)	30 (7.35)
Middle	58 (14.22)	139 (34.07)
High	9 (2.21)	50 (12.25)
Ethnic group		
Oromo	179 (43.87)	209 (51.23)
Other	10 (2.45)	10 (2.45)
Religion		
Muslim	173 (42.40)	206 (50.49)
Other	16 (3.92)	13 (3.19)
Mothers age		
≤25	91 (22.30)	104 (25.49)
26–34	74 (18.14)	91 (22.30)
≥35	24 (5.88)	24 (5.88)
Maternal education		
No education	187 (45.83)	162 (39.71)
Primary and above	2 (0.49)	57 (13.97)
Sex of a child		
Female	86 (21.08)	108 (26.47)
Male	103 (25.25)	111 (27.21)
Age of a child		
6–11	8 (1.96)	33 (8.09)
12–23	40 (9.80)	43 (10.54)
24–35	54 (13.24)	44 (10.78)
36–47	55 (13.48)	41 (10.05)
48–59	32 (7.84)	58 (14.22)
Preceding birth interval		
<24 months	88 (21.57)	66 (16.18)
24–48 months	36 (8.82)	57 (13.97)
>48 months	26 (6.37)	48 (11.76)
1st birth	39 (9.56)	48 (11.76)
ANC attendance		
Yes	63 (15.44)	140 (34.31)
No	126 (30.88)	79 (19.36)
Exclusive breast feeding		
<6 months	12 (2.94)	17 (4.17)
≥6 months	176 (43.14)	201 (49.26)
No EBF	1 (0.25)	1 (0.25)
Frequency of additional food given for the child per 24 h		
≤2 times	73 (17.89)	49 (12.00)
≥3 times	109 (26.72)	163 (39.95)
Not started	7 (1.72)	7 (1.72)
Vaccination		
Yes	156 (38.24)	213 (52.21)
No	33 (8.09)	6 (1.47)
Sign of disease in the past 2 weeks before the survey		
Yes	74 (18.14)	75 (18.38)
No	115 (28.19)	144 (35.29)
Main source of drinking water		
Public pump	145 (35.54)	212 (51.96)
Pond	37 (9.07)	4 (0.98)
Unprotected spring	7 (1.72)	3 (0.74)
Availability of latrine		
Yes	116 (28.43)	180 (44.12)
No	73 (17.89)	39 (9.56)
No. of house rooms		
1–2	159 (38.97)	142 (34.80)
3+	30 (7.35)	77 (18.87)

**Table 7:** Association between underweight and selected socioeconomic variables.

Selected variables	Underweight	
	Underweight frequency (%)	Not underweight frequency (%)
Household family size		
≤5	25 (6.13)	154 (37.75)
≥6	146 (35.78)	83 (20.34)
No. of under-five children		
1	51 (12.50)	109 (26.72)
2	72 (17.65)	109 (26.72)
≥3	48 (11.76)	19 (4.66)
Paternal education		
No education	112 (27.52)	76 (18.67)
Primary and above	59 (14.49)	160 (39.31)
Wealth of household		
Low	119 (29.17)	33 (8.09)
Middle	47 (11.52)	150 (36.76)
High	5 (1.23)	54 (13.23)
Ethnic group		
Oromo	159 (38.97)	229 (56.13)
Other	12 (2.94)	8 (1.96)
Religion		
Muslim	155 (37.99)	224 (54.90)
Other	16 (3.92)	13 (3.19)
Mothers age		
≤25	81 (19.85)	114 (27.94)
26–34	70 (17.16)	95 (23.28)
≥35	20 (4.90)	28 (6.86)
Maternal education		
No education	168 (41.17)	181 (44.36)
Primary and above	3 (0.74)	56 (13.72)
Sex of a child		
Female	74 (18.14)	120 (29.41)
Male	97 (23.77)	117 (28.67)
Age of a child		
6–11	10 (2.45)	31 (7.59)
12–23	36 (8.82)	47 (11.52)
24–35	46 (11.27)	52 (12.74)
36–47	47 (11.52)	49 (12.00)
48–59	32 (7.84)	58 (14.21)
Preceding birth interval		
<24 months	86 (21.07)	68 (16.67)
24–48 months	30 (7.35)	63 (15.44)
>48 months	26 (6.37)	48 (11.76)
1st birth	29 (7.11)	58 (14.22)
ANC attendance		
Yes	51 (12.50)	152 (37.25)
No	120 (29.41)	85 (20.83)
Exclusive breast feeding		
<6 months	13 (3.18)	16 (3.92)
≥6 months	158 (38.72)	219 (53.67)
No EBF	0 (0.00)	2 (0.49)
Frequency of additional food given for the child per 24 h		
≤2 times	74 (18.14)	48 (11.76)
≥3 times	87 (21.32)	185 (45.34)
Not started	10 (2.45)	4 (0.98)
Vaccination		
Yes	139 (34.07)	230 (56.37)
No	32 (7.84)	7 (1.72)
Sign of disease in the past 2 weeks before the survey		
Yes	89 (21.81)	60 (14.71)
No	82 (20.10)	177 (43.38)
Main source of drinking water		
Public pump	130 (31.86)	227 (55.64)
Pond	33 (8.09)	8 (1.96)
Unprotected spring	8 (1.96)	2 (0.49)
Availability of latrine		
Yes	94 (23.04)	202 (49.51)
No	77 (18.87)	35 (8.58)
No. of house rooms		
1-2	147 (36.04)	154 (37.74)
3+	24 (5.88)	83 (20.34)

**Table 8:** Association between wasting and selected socioeconomic variables.

Selected variables	Wasting	
	Wasted frequency (%)	Not wasted frequency (%)
Household family size		
≤5	8 (1.96)	171 (41.91)
≥6	40 (9.80)	189 (46.32)
No. of under-five children		
1	5 (1.22)	155 (37.99)
2	14 (3.43)	167 (40.93)
≥3	29 (7.11)	38 (9.32)
Paternal education		
No education	26 (6.38)	162 (39.80)
Primary and above	22 (5.40)	197 (48.40)
Wealth of household		
Low	30 (7.35)	122 (29.9)
Middle	16 (3.93)	181 (44.36)
High	2 (0.49)	57 (13.97)
Ethnic group		
Oromo	45 (11.03)	343 (84.06)
Other	3 (0.73)	17 (4.17)
Religion		
Muslim	44 (10.78)	335 (82.11)
Other	4 (0.98)	25 (6.13)
Mothers age		
≤25	24 (5.88)	171 (41.91)
26–34	19 (4.66)	146 (35.78)
≥35	5 (1.23)	43 (10.54)
Maternal education		
No education	47 (11.52)	302 (74.02)
Primary and above	1 (0.24)	58 (14.22)
Sex of a child		
Female	21 (5.14)	173 (42.40)
Male	27 (6.62)	187 (45.83)
Age of a child		
6–11	1 (0.24)	40 (9.80)
12–23	8 (1.96)	75 (18.38)
24–35	17 (4.17)	81(19.85)
36–47	12 (2.94)	84 (20.59)
48–59	10 (2.45)	80 (19.61)
Preceding birth interval		
<24 months	35 (8.58)	119 (29.17)
24–48 months	4 (0.98)	89 (21.81)
>48 months	6 (1.47)	68 (16.67)
1st birth	3 (0.73)	84 (20.58)
ANC attendance		
Yes	24 (5.88)	179 (43.87)
No	24 (5.88)	181 (44.36)
Exclusive breast feeding		
<6 months	7 (1.72)	22 (5.39)
≥6 months	41 (10.05)	336 (82.35)
No EBF	0 (0.00)	2 (0.49)
Frequency of additional food given for the child per 24 h		
≤2 times	31 (7.60)	91 (22.30)
≥3 times	7 (1.72)	265 (64.95)
Not started	10 (2.45)	4 (0.98)
Vaccination		
Yes	24 (5.88)	345 (84.56)
No	24 (5.88)	15 (3.68)
Sign of disease in the past 2 weeks before the survey		
Yes	42 (10.29)	107 (26.22)
No	6 (1.47)	253 (62.01)
Main source of drinking water		
Public pump	34 (8.33)	323 (79.17)
Pond	11 (2.69)	30 (7.35)
Unprotected spring	3 (0.73)	7 (1.72)
Availability of latrine		
Yes	14 (3.43)	282 (69.12)
No	34 (8.33)	78 (19.12)
No. of house rooms		
1–2	45 (11.03)	256 (62.74)
3+	3 (0.73)	104 (25.49)

## 2 Methods and materials

Simple random sampling technique was employed to select sample households and children for socioeconomic, demographic, health, environmental and anthropometric data. Only one child was selected from one sample household incase of the presence of more than one child (6–59 months) within a household. Only one under-five child was selected by using simple random sampling when there were more than one under-five children in the household. In case there was no under-five child in a selected household, the next neighboring household was considered. Severely ill child/children was/were excluded and only households having under-five children were included. Socioeconomic data was collected through household survey while anthropometric measurement involved the measurement of weight and height of the under-five children by using the procedure recommended by the World Health Organization (WHO) and United Nations Children׳s Fund (UNICEF) [1], [2] and Central Statistical Agency of Ethiopia [3]. Weight was measured in kilogram to the nearest 0.1 kg by using weighing scale beam (Model QE 2003B). Height/length was measured by using wooden board to the nearest 0.1 cm. For children of age 6–24 months, length was measured horizontally (in a laying position). For children of age over 24 months, height was measured vertically (in a standing position). Two readings were taken and the average was recorded for accuracy. The data were coded and entered in to the computer using EPI info software, exported to ENA for SMART 2011 software and converted into Z-scores of the indices: height-for-age, weight-for-height and weight-for-age. Socioeconomic, demographic, health and environmental data were analyzed by using SPSS Version 20 Software.
