# Supplementary material for: Dataset on child nutritional status and its socioeconomic determinants in Nonno District, Ethiopia
Source: Data Brief. 2017 Jul 11;14:6–14. doi: 10.1016/j.dib.2017.07.007 (PMC5522912; doi:10.1016/j.dib.2017.07.007)
Supplement: Supplementary file 1 — Supplementary material [file mmc1.doc]

**Conflict of Interest Form**

We would like to assure you that there is and/or will not be any conflict of interest related to this data article entitled ‘Child Nutritional Status and Its Socioeconomic Determinants in Ethiopia: Empirical Evidences from Nonno District’.
